# Editorial: Visualization of molecular dynamics in live cells by fluorescent protein-based biosensors

**DOI:** 10.3389/fcell.2022.1054774

**Published:** 2022-10-14

**Authors:** Jihye Seong, Yingxiao Wang

**Affiliations:** ^1^ Brain Science Institute, Korea Institute of Science and Technology (KIST), Seoul, South Korea; ^2^ Division of Bio-Medical Science and Technology, KIST School, Korea University of Science and Technology, Seoul, South Korea; ^3^ Department of Converging Science and Technology, Kyung Hee University, Seoul, South Korea; ^4^ Department of Bioengineering, Institute of Engineering in Medicine, University of California, San Diego, San Diego, CA, United States

**Keywords:** fluorescent protein, fluorescent biosensor, live-cell imaging, FRET, molecular dynamics

Cells continuously respond to a variety of stimulations, such as extracellular ligands, cell-cell interactions, and mechanical signals. These extracellular signals are received by membrane receptors, including ion channels, receptor tyrosine kinases (RTKs), G protein-coupled receptors (GPCRs), integrins, cadherins, and other adhesion molecules (Pierce et al., 2002; Kholodenko, 2006; Horwitz, 2012). They are translated and transferred into the cells through signaling pathways, for example, kinases and phosphatases, second messengers such as cAMP and Ca^2+^, small GTPases, and transcription factors. The spatiotemporal interactions of these signaling molecules control complex cellular processes such as differentiation, cell migration, proliferation, and survival. Genetically encodable fluorescent protein (FP)-based biosensors have been developed for the real-time monitoring of molecular dynamics with the high spatiotemporal resolution, and applied to understand underlying mechanisms of complex cellular processes (Zacharias et al., 2000; Wang et al., 2008; Kim et al., 2021; Dong et al., 2022). Recent advances in the development and applications of the FP-based biosensors are introduced in the current topic entitled *Visualization of molecular dynamics in live cells by fluorescent protein-based biosensors*.

First, Zhou et al. revealed signaling dynamics in T cells after the activation of the chimeric antigen receptor (CAR) specific to the thyroid-stimulating hormone receptor (TSHR). Utilizing fluorescent resonance energy transfer (FRET)-based biosensors, they can observe rapid activation of ZAP70 kinase and ERK in TSHR CAR-T cells upon binding to target cancer cells, thus the efficacy of CAR-T cells can be investigated by FRET imaging. Second, the FRET technology was also applied by Fang et al. to visualize the ERK activation upon cyclic mechanical stretch in airway smooth muscle cells *via* mechanosensitive Ca^2+^ channels. Furthermore, Kim et al. showed that the Piezo1-induced Ca^2+^ influx mediates membrane ruffling and cell survival through PKA, ERK, Rac1, and ROCK activity. Different fluorescent biosensors were applied to investigate various signaling dynamics in live cells, and membrane ruffling was analyzed by spatiotemporal image correlation spectroscopy (STICS). Finally, Kim et al. reviewed a variety of fluorescent biosensors with different sensing strategies such as bioluminescence resonance energy transfer (BRET), FRET, circular permuted FP (cpFP), and nanobody, for the real-time monitoring of each stage of GPCR activation. Thus, a broad range of dynamic signaling events in response to different extracellular stimuli can be monitored and tracked by live-cell imaging with advanced fluorescent biosensors.

The dynamic molecular interactions are tightly controlled in space and time. Thus, it is critical to monitor the real-time molecular events by fluorescent biosensors in subcellular regions. Ku et al. monitored the movement of chromatin in the nucleus by single-particle tracking of CRISPR/dCas9-tagged FP in live cells and reported the effects of transcription-dependent physical perturbations on the chromosome dynamics. In addition, Karasev et al. compared different nuclear localization signals (NLSs) with different importin specificities and identified uncommon NLSs optimized for neurons. They utilized these NLSs to develop an optogenetic tool for the nuclear export of proteins. Kim et al. reviewed the optimization processes for the development of FRET-based biosensors and discussed the available subcellular targeting sequences for fluorescent biosensors. These localization sequences include nuclear export signal (NES), NLS, mitochondrial targeting sequence, the signals for membrane microdomains and outside plasma membrane, and ER retention sequence. The correct subcellular localization of fluorescent biosensors is critical for the sensitive monitoring of subtle but essential physiological molecular events.

It has been significant efforts for the development of enhanced FPs and new fluorescent biosensors with diverse colors. Rad et al. explored the residues of a voltage-sensing domain to increase voltage sensitivity of the current genetically encoded voltage indicators (GEVIs) and developed Plos6-vs with improved dynamic range and faster response kinetics. Wu et al. reviewed fluorescent indicators for detecting monatomic ions such as Ca^2+^, Zn^2+^, K^+^, Na^+^, H^+^, Cl^−^, Cu^+^, and toxic ions. They introduced various sensing strategies utilizing different colors of FPs, such as red, far-red, and near-infrared (NIR). Matlashov et al. developed a genetically encoded calcium indicator (GECI) based on FRET between miRFP670nano and miRFP720, iGECInano, which shows enhanced brightness and photostability and faster response kinetics. This NIR GECI allows spectral crosstalk-free combinations with green-colored biosensors. Finally, Ning et al. introduced Crimson, a bright, nontoxic, and non-aggregating red FP. The membrane-targeted Crimson-CAAX allows long-term labeling of thin neurites, dendritic spines, and filopodia in neurons and can be applied together with green-colored probes. The multicolor fluorescent biosensors will allow a deeper understanding of the complex molecular dynamics of multiple signaling events.

In summary, the current topic, composed of eight research articles and three review articles, introduces recent progresses in FP-based biosensors and their applications for the visualization of spatiotemporal molecular dynamics in live cells. The further development of advanced biosensor technologies and their applications in live-cell imaging will pave the way for the future discovery of the underlying mechanisms of complex molecular dynamics crucial for cell functions.
